# Inequalities in caesarean section in Burundi: evidence from the Burundi Demographic and Health Surveys (2010–2016)

**DOI:** 10.1186/s12913-020-05516-8

**Published:** 2020-07-14

**Authors:** Sanni Yaya, Betregiorgis Zegeye, Dina Idriss-Wheeler, Gebretsadik Shibre

**Affiliations:** 1 School of International Development and Global Studies, University of Ottawa, 120 University Private, Ottawa, Ontario K1N 6N5 Canada; 2 The George Institute for Global Health, The University of Oxford, Oxford, UK; 3 HaSET Maternal and Child Health Research Program, Shewarobit Field Office, Shewarobit, Ethiopia; 4 Interdisciplinary School of Health Sciences, University of Ottawa, Ottawa, Ontario Canada; 5 Department of Reproductive, Family and Population Health, School of Public Health, Addis Ababa University, Addis Ababa, Ethiopia

**Keywords:** Caesarean section, Inequality, Global health, Burundi, DHS

## Abstract

**Background:**

Despite caesarean section (CS) being a lifesaving intervention, there is a noticeable gap in providing this service, when necessary, between different population groups within a country. In Burundi, there is little information about CS coverage inequality and the change in provision of this service over time. Using a high-quality equity analysis approach, we aimed to document both magnitude and change of inequality in CS coverage in Burundi over 7 years to investigate disparities.

**Methods:**

For this study, data were extracted from the 2010 and 2016 Burundi Demographic and Health Surveys (BDHS) and analyzed through the recently updated Health Equity Assessment Toolkit (HEAT) of the World Health Organization. CS delivery was disaggregated by four equity stratifiers, namely education, wealth, residence and sub-national region. For each equity stratifier, relative and absolute summary measures were calculated. We built a 95% uncertainty interval around the point estimate to determine statistical significance.

**Main findings:**

Disparity in CS was present in both survey years and increased over time. The disparity systematically favored wealthy women (SII = 10.53, 95% UI; 8.97, 12.10), women who were more educated (PAR = 8.89, 95% UI; 8.51, 9.26), women living in urban areas (D = 12.32, 95% UI; 9.00, 15.63) and some regions such as Bujumbura (PAR = 11.27, 95% UI; 10.52, 12.02).

**Conclusions:**

Burundi had not recorded any progress in ensuring equity regarding CS coverage between 2010 and 2016. It is important to launch interventions that promote justified use of CS among all subpopulations and discourage overuse among high income, more educated women and urban dwellers.

## Background

Cesarean section (CS) is a necessary element of emergency obstetric care services [1], as it can successfully prevent both maternal and childhood death and morbidity [2]. However, many women are electing to give birth through CS without medical justification to do so [3]. Although at times necessary, there is evidence of considerable risk from CS to the mother and infant [4, 5], and therefore, only woman who actually require CS should undergo the procedure [3, 5]. This approach helps avoid the complications of CS which can lead to unintended deaths of mothers and infants.

Universal coverage and utilization of CS is not the aim of interventions; it is the challenge of determining a suitable optimum level of CS at a population level that will ensure those who needed it, actually receive the procedure, and prevent its unjustified use [1]. Lead by the World Health Organization (WHO) in 1985, key stakeholders discussed the appropriate technology for birth and delivered recommendations indicating that optimal rates of CS should not be higher than 10–15% at the population level [6]. Since then, governments and clinicians around the world expressed alarm at the increase in the number of CSs around the world, with its use almost doubling between 2000 and 2015 from 16 million to 29.7 million [7].

A systematic review by Betran et al. (2015) suggested an unadjusted optimal rate for CS between 9 and 16%; when adjusting for socio-economic factors, the asssociation between CS rates and decrease in maternal, newborn and infant mortality disappears [8]. Thus, rates below the threshold mean other factors and not the CS rate maybe the determinants of mortality (i.e. access to health facility and skilled healthcare) and usually an issue in LMICs; CS rates above the threshold are not reducing maternal mortality, and maybe unjustified caesreans [7]. The 2017 Guide to Managing Complications in Pregnancy and Childbirth by the WHO, UNICEF and UNFPA suggest 15% of a population may develop life-threatening complications and may require skilled care and invasive procedures such as CS [9]. In high-income countries, an optimal level seems to lie anywhere between 6 to 8% [10]. In lower resourced settings (i.e. sub-Saharan Africa and Southern Asia) extremely low rates of caesarean section were found among the very poor, regardless of increased national rates, indicating inequalities within countries are found in specific sub-groups [10]. As part of the Lancet series on optimizing casesrean section use, Boerma et al. (2018) indicated that CS use was almost five times more frequent in births in richest versus the poorest quintiles in LMICs; markedly high use of CS was observed among low obstretric risk births (i.e. to educated women) and it was used 1.6 times more frequently in private versus public facilities [7].

Assessing the overall rate or coverage of a health care service indicator such as caesarean section service delivery is useful for investigations of national and global trends. However, examining within country disparity of the indicator is useful to fight against and eliminate the unjustified inequalities across the different population groups within a country. Under the ambitious Sustainable Development Goals (SDGs), ensuring the universal coverage of reproductive, maternal and child health care services, including CS, is one of the 17 targets that should be attained by 2030 [11]. It is not enough for coverage of caesarean service delivery to reach an optimal level in a country’s population because aggregate numbers, as discussed, offer an incomplete story. There is no guarantee of caesarean service to all population groups who need it [8]. This is especially important in countries where there is high inequality in health care services. For this study, it is of utmost importance to study CS delivery inequality following the rigorous approach recommended by the WHO for health equity studies [1].

In 2015, and based on data extracted from 169 countries, between 20 to 22.4% of births were CS; this was nearly double the number of CS births which took place in 2000 [8]. Substantial inequalities in use of CS were seen within and between countries [8, 11–15]. Latin American and Caribbean regions had the highest CS birth compared to West Africa and Central Africa [3]. Africa has the lowest coverage for CS [11], and particularly in Sub-Saharan Africa (SSA), CS had not increased over the last 20 years while at the same time, there were increases in CS in other parts of the world [13]. When CS was disaggregated by different population groups in a country, high income [11–13, 15, 16] and more educated women [13, 16] and urban dwellers were more likely to use the service than women living in rural settings [15]. Similar to other SSA countries, Burundi had one of the lowest utilization of the caesarean delivery in the world; between 8.8 and 11% of babies born via CS [17]. This aggregate level does not provide insight into the socio-economic factors that affect the rate of caesarean delivery taking place in Burundi. Understanding the association of CS rates and outcomes (i.e. mortality and morbitidy) at the sub-national level across different sub-populations provides more valuable data to assess and make decisions regarding interventions to reduce maternal mortality in relation to lack of appropriate maternal health services and skilled personnel.

Inequality analysis of CS is useful to generate evidence about population subgroups within country; in Burundi, there is little information on this issue using rigorous and well-established approaches. A 2016 study looked at maternal outcomes 2 years following emergency c-section in rural Burundi concluding that despite encouraging maternal outcomes, innovative ways of promoting family planning 24 months in this vulnerable group is needed [18]. Work on disparities in CS prevalence and determinants across sub-Saharan Africa indicated that in 2011, 11% of CSs in Burundi took place in private healthcare settings while 8.8% were in public healthcare; the conclusions were based on the pooled data for the region providing insight into heath policies to improve outcomes of CS care [16]. A multi-country study, part of which was looking at CS rates and indications in Kabezi in Burundi, revealed a national CS rate of 4.1% among the studied population between 2010 and 2011 [19]. Within country inequalities in CS rates across 72 LMICs provided some information regarding Burundi (i.e. national average of 4.3% using 2010 BDHS); but only with regards to economic status (i.e. a difference of 6.5 percentage points between richest [8.8%] and poorest [4.3]) and place of residence (i.e. the CS average was 13.2% [CI 95%; 10.4, 16.7] for urban and 3.5% [CI 95%; 2.9,4.4] for rural) [10].

High-quality and within country, sub-national level evidence is necessary to help reduce the unjustified gap between various population groups and ensure reasonable access of the service to all who need it. In this study, we aimed to comprehensively investigate both extent and time trends of inequalities in use of CS in Burundi using two series of Burundi Demographic and Health Surveys (BDHS) in the World Health Organization (WHO) Health Equity Assessment Toolkit (HEAT) Software.

The WHO HEAT application is a software that explores and compares inequality across five dimensions: economic status, education, place of residence, sex and subnational region [20]. This enables users to investigate the health inequality in one setting as well as compare it to a situation in other settings across the dimensions [20–22]. The functionality and procedures of how it works are described in detail elsewhere [20]. In brief, WHO developed the application in 2016 in an attempt to assist researchers, policy makers as well as planners with an easy application to assess and monitor health inequalities over time by investigating certain socio-economic factors [22]. This software helps investigate, explore and benchmark disparities on 30 reproductive, maternal, newborn, and child health (RMNCH) indicators within and between country; a key indicator being caesarian section [20–22].

## Methods

### Study area

Home to over 11 million people, Burundi is the third most densely populated country in sub-Saharan Africa (SAA) with an estimated 463 inhabitants per km^2^ [23], and an increasing population that is expected to double by 2040 [18]. Plagued by political uncertainty and violence, the country is poor and severely fragile in terms of its security, economy, society, politics and environment [24]. Although the under-five child mortality rate in Burundi has gradually decreased from 156.4 deaths per 1000 live births in 2000 to 58.5 deaths per 1000 in 2018 [25], it is still over twice the target of less than 25 per 1000 set by the UN 2030 Agenda [26]. The maternal mortality ratio per 100,000 live births has also dropped in Burundi over the past two decades from 1010 deaths per 100,000 live births in 2000 to 538 deaths per 100,000 live births in 2017 and still above the UN 2030 Agenda target of less than 70 deaths per 100,000 by 2030 [26, 27]. Although under-five child and maternal mortality rates have improved over the past two decades in Burundi, they continue to be higher than target indicators [28]. Furthermore, the 2015 political crisis hampered service delivery, particularly affecting maternal and child health services during this time [28]. Significant disparities in coverage and utilization due to socioeconomic status (i.e. financial barriers) and access (i.e. rurality, transport) to maternal and child health services continue in Burundi [28].

### Data sources

The 2010 and 2016 Burundi Demographic and Health Surveys (BDHS) were used in this study. DHS uses a stratified two-stage cluster design where the first stage includes Enumeration Areas (EA) selected through a Probability Proportional to Size approach where large more representative EAs have a higher chance of being in the sample than the small EAs. In the second stage, a random sample of households are drawn from the selected EAs. The household surveys collected data on maternal reproductive and child health in Burundi representative at the national, residence and regional level [29]. Even though the BDHS was carried out in 1987, 2010 and 2016, the 1987 BDHS is not available in the WHO HEAT software; therefore, we confined our analysis to the 2010 and 2016 rounds. Detailed information regarding the BDHS study design are published elsewhere for 2010 [30] and 2016 [31]. A total sample of 24,520 women participated in the 2010 BDHS, of whom 16,778 had a birth in the last 5 years, and 375 births were through CS while remaining 7323 did not have a CS [30]. In 2016, a total sample of 45,419 women were surveyed, of whom 32, 312 had birthed a child and answered the question whether the child was born by caesarean section; 786 had a caesarean and 12,321 did not have a caesarian [31].

### Variables and measurements

CS is defined as the percentage of births delivered by caesarean section among all live births in the 5 years prior to the surveys for the last birth and the next-to-last birth. The question asked: “Was (name) delivered by caesarean, that is, did they cut your belly open to take the baby out?”; the answer options provided were “yes” or “no” [30, 31]. CS was the outcome variable of interest and inequality was measured according to the four equity stratifiers: economic status, education, place of residence, and subnational region. Economic status was approximated through wealth index and is classified into five quintiles: poorest, poor, middle, rich and richest. Wealth index is a standard socioeconomic variable in the DHS and we used the variable as it is. Using a statistical data reduction technique, *Principal Component Analysis*, wealth index is computed based on household assets and possessions following methods introduced by the Rutstein SO and Johnson K [5]. Educational status is categorized as no education, primary, secondary or higher; place of residence as urban versus rural, and the sub-national region included five and 18 regions in 2010 and 2016 surveys, respectively. Education and wealth have a natural ordering and are known as ordered equity stratifiers whereas place of residence and regions are non-ordered equity stratifiers. The type of summary measures to be calculated are partly determined by whether or not the equity stratifiers are ordered or not [21].

### Statistical analysis

Using the recently updated WHO’s HEAT software (2019 update) [22], we analyzed the socioeconomic and area-based CS disparities. The inequality analysis was completed by, first, disaggregating CS according to the above-mentioned dimensions of inequality and, subsequently interpreting the findings derived from summary measures. Then, we calculated absolute inequality summary measures that included Difference (D), Population Attributable Risk (PAR), and Slope Index of Inequality (SII). These measures were calculated for the four equity stratifiers; for the wealth and education dimensions of inequality, we calculated all of the three inequality measures, and for the region and place of residence, we only calculated the D and PAR.

The detailed methods regarding calculation and interpretation of the measures used in the study have been detailed elsewhere [21]. Since CS is a favorable indicator (i.e. a lifesaving measure), positive values of a measure show disproportionate use of the service among the advantaged sub-groups, while negative values indicate that disadvantaged groups are using the service most. The higher their absolute value, the greater the inequality. Zero value for the measures show absence of inequality.

D is a simple measure that shows absolute difference between two categories. The other two (PAR, SII), on the other hand, are weighted complex measures of inequality and take into account sizes of all the subpopulations used in the calculation, thereby producing more robust estimates that could represent the entire subpopulation [20, 21]. SII was computed for education and wealth equity stratifiers as it requires an ordered variable. Each point estimate is accompanied by an Uncertainty Interval (95%UI) to identify CS disparities that are statistically significant and to determine whether or not the inequality changed with time. For all inequality measures, the lower and upper bounds of the UI must not contain zero for CS inequality to exist. We assessed the trend of inequality for each summary measure by referring to the UIs for the different survey years; if the UIs did not overlap, inequality existed. The findings were presented per the recommendation of the Strengthening Reporting of Observational studies in Epidemiology (STROBE) reporting guidelines [32].

### Ethical considerations

The demographic health surveys are available publicly and ethics approvals were completed by institutions that commissioned, funded, and managed the surveys. DHS surveys are approved by Inner City Fund (ICF) International and an in-country Institutional Review Board (IRB) to ensure protocols are in compliance with the U.S. Department of Health and Human Services regulations for the protection of human subjects.

## Results

Table 1 shows birth by caesarean section across categories of each subpopulation of economic, education and area-based dimensions in two survey rounds along with their sample size. Except in quintile 5 (richest) which shows significantly higher CS coverage, approximately similar coverage was seen in all other wealth quintiles in 2010. Similar CS service coverage was seen in 2016 among quintiles 1 and 2, and quintiles 3 and 4, however, the highest coverage was seen in the richest sub-population. With the exception of quintile 4 and 5, which saw significantly increasing coverage, a constant pattern was seen in other wealth quintiles over the course of 6 years.

**Table 1:** Coverage of birth by caesarean section across wealth quintiles, educational status, place of residence and subnational national region in Burundi from 2010 to 2016

Dimension of Inequality	Subgroup	2010		2016	
		Estimate (95% UI)	Pop^**n**^	Estimate (95% UI)	Pop^**n**^
Economic status	Quintile 1 (poorest)	3.01 (1.98, 4.54)	1618	2.56 (1.91, 3.44)	3064
	Quintile 2	3.30 (2.34, 4.65)	1669	2.96 (2.09, 4.18)	2920
	Quintile 3	3.14 (2.15, 4.56)	1676	3.84 (3.03, 4.86)	2769
	Quintile 4	2.61 (1.75, 3.87)	1590	4.97 (3.91, 6.28)	2582
	Quintile 5 (richest)	8.28 (6.70, 10.18)	1426	12.53 (10.67, 14.66)	2274
Education	No education	2.83 (2.21, 3.62)	4178	3.93 (3.27, 4.72)	6396
	Primary school	4.38 (3.46, 5.52)	3312	3.99 (3.36, 4.73)	5753
	Secondary school or higher	10.75 (8.18, 14.00)	489	13.92 (11.91, 16.21)	1461
Place of residence	Rural	3.23 (2.66, 3.90)	7322	3.91 (3.41, 4.47)	12,367
	Urban	12.13 (9.54, 15.29)	657	16.23 (13.21, 19.77)	1242
Sub - national Region	Bujumbura [Bubanza^a^]	12.92 (9.63, 17.11)	400	7.72 (5.09, 11.56)	753
	North [Bujumbura Rural^a^]	3.57 (2.62, 4.83)	2396	5.82 (3.96, 8.49)	761
	Centre-east [Bururi^a^]	4.01 (2.89, 5.52)	1986	6.95 (4.52, 10.54)	367
	West [Cankuzo^a^]	3.19 (2.04, 4.96)	1576	3.18 (1.86, 5.40)	442
	South [Cibitoke^a^]	3.01 (2.15, 4.20)	1620	6.30 (4.79, 8.25)	842
	Gitega^a^	NA	NA	5.02 (2.90, 8.57)	1109
	Karusi^a^	NA	NA	0.97 (0.54, 1.73)	772
	Kayanza^a^	NA	NA	3.85 (2.73, 5.41)	814
	Kirundo^a^	NA	NA	3.01 (1.86, 4.81)	1088
	Makamba^a^	NA	NA	6.21 (4.40, 8.69)	778
	Muramvya^a^	NA	NA	3.56 (1.71, 7.25)	472
	Muyinga^a^	NA	NA	1.54 (0.65, 3.62)	1188
	Mwaro^a^	NA	NA	4.22 (2.53, 6.94)	400
	Ngozi^a^	NA	NA	4.97 (3.41, 7.19)	1067
	Rutana^a^	NA	NA	5.08 (2.74, 9.23)	562
	Ruyigi^a^	NA	NA	3.81 (2.43, 5.93)	763
	Bujumbura Mairie^a^	NA	NA	16.31 (11.46, 22.67)	661
	Rumonge^a^	NA	NA	5.66 (3.80, 8.36)	764
National average		3.964269		5.035336	

^a^ indicate regions in 2016 survey, *NA* Not Applicable for 2010 survey since there were only five regions

In 2010, the distribution of CS birth was highest in secondary or higher education group followed by primary and no education subpopulations. In 2016, a slightly different pattern of CS was observed in relation with education; it was highest among the women who completed secondary or higher education, but it was the same among the no education and primary education groups. This happened since CS among mothers with no education increased with time whereas it remained stagnant among the primary education groups. There was a larger increased in CS among women who had finished secondary or higher schooling between 2010 and 2016 compared to the other two groups. The coverage of CS birth was significantly higher among urban residents in both surveys with an overtime increasing pattern in both.

Regarding subnational regions, we were only able to comment on the dynamics of region-based CS for five regions that had CS estimates from 2010 to 2016. While Bujumbura rural, Bururi, and Cibitoke had increasing trends of CS, Bubanza showed a decreasing trend, and CS in Cankuzo did not change with time. See Table 1 for details.

### CS inequalities by summary measures

Table 2 shows degree of socio-economic and area-based inequalities in CS birth and how it evolved over time in Burundi between 2010 and 2016. In both studied survey periods, substantial CS disparities were observed in all the dimensions of inequality - the poor, women without formal education, rural residents and certain regions suffered inequitable coverage. The overtime dynamics showed that the disparity became worse.

**Table 2:** Time trends of CS inequalities by wealth, education, residence and region in Burundi, 2010 to 2016 DHSs

Dimensions of inequalities	Summary measures	2010	2016
		Estimate (95%UI)	Estimate (95%UI)
Economic status	D	5.26 (3.14, 7.39)	9.96 (7.84, 12.09)
	PAR	4.31 (3.54, 5.09)	7.50 (6.97, 8.03)
	SII	4.56 (2.93, 6.18)	10.53 (8.97, 12.10)
Educational status	D	7.91 (4.95, 10.88)	9.98 (7.72, 12.25)
	PAR	6.78 (6.39, 7.18)	8.89 (8.51, 9.26)
	SII	5.75 (3.92, 7.57)	7.13 (5.60, 8.65)
Place of residence	D	8.90 (5.98, 11.82)	12.32 (9.00, 15.63)
	PAR	8.16 (7.96, 8.37)	11.19 (11.01, 11.37)
Subnational region	D	9.90 (6.06, 13.74)	15.34 (9.74, 20.93)
	PAR	8.95 (8.18, 9.73)	11.27 (10.52, 12.02)

*D* Difference, *PAR* Population Attributable Risk, *SII* Slope Index of Inequality

For economic status, the Difference (D), PAR and SII demonstrated the statistically significant and disproportionate use of CS among the advantaged sub-groups (i.e. richest quintile). Furthermore, the UIs across all three do not overlap, therefore indicating that the overtime increase in disparity is also significant. For educational status, all three summary measures showed that more women with secondary or higher education underwent CS procedures compared to women with no education. PAR indicated a significant increase between 2010 and 2016 from 6.78 (95% UI; 6.39, 7.18) to 8.89 (95% UI; 8.51, 9.26), respectively. Inequality in place of residence was apparent with both D and PAR, the advantage of CS use was for women in urban regions. PAR demonstrated a significant increase in disparity over time from 8.16 (95% UI; 7.96, 8.37) in 2010 to 11.19 (95% UI, 11.01,11.37). Finally, there was sub-national regional disparity as seen by both the D and the PAR summary measures; the disparity overtime increased as demonstrated by PAR. Bujumbura (in 2010) and Bujumbura Mairie (in 2016) had the highest rates of CSs, and the disparity compared to the South in 2010 and Karusi in 2016 flags the need to investigate sub-national inequalities in CS coverage.

## Discussion

The systematic investigation of CS inequality in Burundi over the course of 7 years was prompted by the need to generate high-quality evidence. Disaggregating health care interventions such as CS by relevant equity stratifiers has been one of the fundamental sustainable development goals since 2015 [1]. It is only when all population groups in a country are targeted with an intervention of interest that the Sustainable Development Goal (SDG) tenant of “leaving no one behind” is attained. The WHO continues to work on defeating persistent in-country inequalities in health care service provision. It developed and released an equity analysis toolkit that researchers around the world can use to generate reliable, comparable evidence useful for improving equity nationally and globally. For this study, we used the recently updated offline version of the HEAT that incorporates more recent DHS and Multiple Indicator Cluster Surveys (MICS) [22]. Our finding that wealthier women disproportionately use CS is in line with prior evidence [11–13, 15, 16]. Burundi is one of the poverty hit countries in Africa, with an estimated 1.77 million people requiring humanitarian assistance [33]. Reducing poverty in the country could facilitate utilization of lifesaving interventions such as CS.

The educational inequality in use of CS favored women with secondary or higher education albeit the gap is not very wide. In 2017, Burundi had a literacy rate of 68.4% among those 15 years or older, with the female literacy rate falling behind that of males at 61.2% [34]. This translates to nearly 40 women (15 years or older) in 100 who cannot read and write. If the illiterate and primary education groups were on par with the secondary or higher education group in terms of CS use, then Burundi would have increased the national average CS coverage by seven and nine percentage points in 2010 and 2016, respectively. The finding in our study that CS concentrates more among the educated mothers is in concordance with previous work [13, 16].

Urban areas disproportionately obtain CS service compared to rural settings, with the PAR showing increasing trend between the two series of DHS. The fact that urban places utilize more of the service than rural counterparts is supported by existing evidence [15]. In both urban and rural areas, CS had seen a slight increment over the 7-year period; however, the pace of rise in CS among the urban area was faster than among rural places, and this explains the overtime widening of the urban-rural gap in CS that is skewed towards urban areas. Similarly, there was substantial subnational regional variation in use of CS with some regions such as Bujumbura and Center East receiving most of the service than other regions in the country. Subsequently, this supports previous findings that showed distribution of caesarean delivery that is skewed towards urban settings [15].

The observed CS delivery disparity implies that poor, non-educated and rural living women are accessing CS that is far lower than the optimal rate required to combat excess deaths associated with obstetric complications [9, 10]. WHO recommends that the maximum optimal value of CSs should not exceed 15% in a population [9]. In our study, except in urban settings and Bujumbura Mairie in 2016 where the average CS was around 16%, even the rich and educated women did not use the service up to the recommended level of 15% in either survey years, suggesting underutilization of the service. This is worrying because the less than optimal level of national coverage of CS and the variation in coverage between subgroups could lead to maternal deaths in the country that could be averted by the surgical procedure. The aggregate CS level of 15% or more at a population level does not tell the whole story because it does not necessarily indicate whether women in all subpopulation groups and those in need of CS are actually accessing the service in a country. Furthermore, optimal rates provide no indication of why some have easy (and unnecesary) access to CS, while others who may need it do not. In a Lancet series on optimizing caesarean section use, Betran et al. (2018) provide evidence-based insight to the drivers of excessive CS use that range from complex factors related to childbearing women, their families, and community (i.e. labour pain, pelvic floor damage); the health professionals and their influence on the decision about mode of birth (i.e. logistical and financial incentives; fear of litigation); and the healthcare systems along with financial reimbursements, organizational design, culture, and settings [35]. In the same series, Boerma et al. (2018) describe the frequency of, trends in, determinants of and inequalities in CS globally, ultimately suggesting that within and across country disparities in CS were large [7]. The frequency of CS births was among the richest quintiles versus the poorest in the LMICs and educated women who have access to, and ability to pay for, private facilities. Furtheremore, there was global increase of CSs ocurring in health facilities along with increased use of CSs in health facilities [7]. In concordance with our findings, poor and vulnerable women do not have access to life saving surgery during childbirth that include issues of location of health facilities and shortatges of a skilled health workforce [7, 27].

This study highlighted, and demonstrated, the need to generate information on CS disaggregated by the relevant equity stratifiers to explore extent of CS use in key population groups. This helps policy makers separate out subgroups that do use the service more than the agreed CS level and to discourage them from over using it. Even if CS is an effective intervention in averting deaths associated with obstetric complications when necessary medically [2], it poses risks to woman and infants when used without medical necessity [4, 5].

WHO recommends that CS should be practiced only if medically necessary, and health facilities should avoid aiming for a certain level of CS, and instead, perform the procedure when it is indicated medically [3]. At the same time, disaggregated information about CS is useful to identify subgroups that are underusing the service or there is insufficient coverage of and accessibility to the service when necessary. If coverage of CS increases among the disadvantaged subgroups, the overall national coverage of the service could be improved which, in turn, positively contributes to the 2030 relevant SDG targets. In fact, data on disaggregation have been the central element of the 2030 global goal [1].

### Strengths and limitations of the study

The strength of the study was that CS inequality was analyzed by calculating different measures of inequality which allowed exploration of disparity from wider perspectives. Doing so is important in overcoming the limitations of one method by the strength of the other method. The study also presented inequality findings with respect to four dimensions of inequality which can assist governments by identifying where and how to strengthen their efforts towards realization of the equity-oriented SDG targets in relation to maternal health. Finally, the study used the high-quality data available through the well-established WHO health equity monitor database contributing to the quality of the conclusions drawn from the study. However, the study has some limitations. Our analysis could not provide an in-depth assessment of problems that led to the observed CS disparities, making it challenging for decision makers to put in place targeted interventions. The study design cannot account for factors leading to changes observed over the 6-year period between the two surveys. Furthermore, the Caesarean Section measure used in the study does not distinguish between elective or medically necessary procedures; answers are based on self-reports. Future studies need to apply a decomposition method to better appreciate the level of influences of common problems on the observed CS disparity. Finally, the study presented inequality of the service at sub-regional levels, we may also benefit from additional studies comparing CS disparities at the health facility level.

## Conclusions

Burundi experienced disparity in use of CS, which increased over time between 2010 and 2016. Our findings indicate that more work needs to be done to ensure that all subpopulations that require medically necessary CS are able to access the service in order to avoid unnecessary maternal and infant deaths. It is also important to discourage unjustified use of CS among certain subgroups to reduce deaths associated with unnecessary CS delivery.

## Data Availability

The datasets generated and/or analyzed during the current study are available in the WHO’s HEAT version 3.1 [https://www.who.int/gho/health_equity/assessment_toolkit/en/].

## Abbreviations

D: Difference
CS: Caesarean section
BDHS: Burundi Demographic and health Survey
HEAT: Health Equity Assessment Toolkit
PAR: Population Attributable Risk
PPS: Probability Proportional to Size
WHO: World Health Organization
SDG: Sustainable Development Goal
SII: Slope Index of Inequality

## References

[1] World Health Organization, UNFPA, UNICEF, AMDD. Monitoring emergency obstetric care:a handbook. Geneva: WHO; 2009. Available at http://whqlibdoc.who.int/publications/2009/9789241547734_eng.pdf?ua=1. Accessed on 29 Apr 2020.

[2] Hannah ME, Hannah WJ, Hewson SA, Hodnett ED, Saigal S, Willan AR, Term Breech Trial Collaborative Group (2000). Planned caesarean section versus planned vaginal birth for breech presentation at term: a randomised multicentre trial. Lancet.

[3] WHO. WHO Statement on Caesarean Section Rates. Every effort should be made to provide caesarean sections to women in need, rather than striving to achieve a specific rate. Accessed 18 Oct 2019.

[4] Sandall J, Tribe RM, Avery L, Mola G, Visser GHA, Homer CSE (2018). Short-term and long-term effects of caesarean section on the health of women and children. Lancet.

[5] Lumbiganon P, Laopaiboon M, Gulmezoglu AM, Souza JP, Taneepanichskul S, Ruyan P, et al. Method of delivery and pregnancy outcomes in Asia: the WHO global survey on maternal and perinatal health 2007–08. Lancet. 2010 ;375(9713):490–9. 10.1016/S0140-6736(09)61870-5 20071021

[6] WHO (1985). Appropriate technology for birth. Lancet..

[7] Boerma T, Ronsmans C, Melesse DY, Barros AJD, Barros FC, Juan L (2018). Global epidemiology of use of and disparities in caesarean sections. Lancet..

[8] Betran AP, Torloni MR, Zhang J, Ye J, Mikolajczyk R, Deneux-Tharaux C, et al. What is the optimal rate of caesarean section at population level? A systematic review of ecologic studies. Reprod Health. 2015. p. 12. Available from: https://www.ncbi.nlm.nih.gov/pmc/articles/PMC4496821/. 10.1186/s12978-015-0043-6 PMC4496821 26093498

[9] World Health Organization (WHO). Managing complications in pregnancy and childbirth: a guide for midwives and doctors [Internet]. Maternal Child Survival Program. 2nd ed. Geneva: World Health Organization; 2017. Licence: CC BY-NC-SA 3.0 IGO. [cited 2020 Jul 10]. Available from: https://www.mcsprogram.org/resource/managing-complications-pregnancy-childbirth-guide-midwives-doctors/.

[10] UCSF health. High-Risk Pregnancy. Available from: https://www.ucsfhealth.org/conditions/high-risk_pregnancy/. Accessed 29 Apr 2020.

[11] Boatin AA, Schlotheuber A, Betran AP, Moller A-B, Barros AJD, Boerma T, et al. Within country inequalities in caesarean section rates: observational study of 72 low and middle income countries. BMJ [Internet]. 2018;360:k55. 10.1136/bmj.k55. 10.1136/bmj.k55 PMC5782376 29367432

[12] El-Khoury M, Hatt L, Gandaho T. User fee exemptions and equity in access to caesarean sections: an analysis of patient survey data in Mali. Int J Equity Health. 2012;11:49. 10.1186/1475-9276-11-49 PMC3480938 22931249

[13] Klemetti R, Che X, Gao Y, Raven J, Wu Z, Tang S, et al. Cesarean section delivery among primiparous women in rural China: an emerging epidemic. Am J Obstet Gynecol. 2010;202(1):65.e1-6. 10.1016/j.ajog.2009.08.032. 10.1016/j.ajog.2009.08.032 19819416

[14] Ronsmans C, Holtz S, Stanton C (2006). Socioeconomic differentials in caesarean rates in developing countries: a retrospective analysis. Lancet..

[15] Cavallaro FL, Cresswell JA, França GVA, Victora CG, Barrosb AJD, Ronsmansa C (2013). Trends in caesarean delivery by country and wealth quintile: cross-sectional surveys in southern Asia and sub-Saharan Africa. Bull World Health Organ.

[16] Gebremedhin S. Trend and socio-demographic differentials of Caesarean section rate in Addis Ababa, Ethiopia: analysis based on Ethiopia demographic and health surveys data. Reprod Health. 2014;11(1):14. 10.1186/1742-4755-11-14 PMC3925324 24563907

[17] Yaya S, Uthman OA, Amouzou A, Bishwajit G. Disparities in caesarean section prevalence and determinants across sub-Saharan Africa countries. Glob Health Res Policy. 2018;3(1):19. Available from: 10.1186/s41256-018-0074-y. 10.1186/s41256-018-0074-y PMC6027740 29988650

[18] van den Boogaard W, Manzi M, De Plecker E, Caluwaerts S, Nanan-N'zeth K, Duchenne B (2016). Caesarean sections in rural Burundi: how well are mothers doing two years on?. Public Health Action.

[19] Chu K, Cortier H, Maldonado F, Mashant T, Ford N, Trelles M (2012). Cesarean section rates and indications in sub-Saharan Africa: a multi-country study from Medecins sans Frontieres. PLoS One.

[20] Hosseinpoor AR, Nambiar D, Schlotheuber A, Reidpath D, Ross Z. Health Equity Assessment Toolkit (HEAT): software for exploring and comparing health inequalities in countries. BMC Med Res Methodol [Internet]. 2016;16(1):141. Available from: 10.1186/s12874-016-0229-9. 10.1186/s12874-016-0229-9 PMC5069829 27760520

[21] World Health Organization. Handbook on health inequality monitoring with a special focus on low and middle income countries. Geneva: World Health Organization; 2013. Available from: http://www.who.int/gho/health_equity/handbook/en/. Accessed on 29 Apr 2020.

[22] World Health Organization (2019). Health Equity Assessment Toolkit (HEAT): Software for exploring and comparing health inequalities in countries. Built-in database edition. Version 3.1.

[23] United Nations (UN) Department of Economic and Social Affairs. Population Dynamics. World Population Prospects 2019. Available from https://population.un.org/wpp/. Accessed on 13 May 2020.

[24] Organization for Economic CO-operation and Development (OECD). States of Fragility 2018. Available from http://www.oecd.org/dac/states-of-fragility-2018-9789264302075-en.htm. Accessed on 13 May 2020.

[25] Burundi (BDI) - Demographics, Health & Infant Mortality. 2020. Available from: Available from: https://data.unicef.org/country/bdi/. [cited cited 2020 Jul 2].

[26] United Nations General Assembly (2015). Transforming our world: the 2030 agenda for sustainable development.

[27] Maternal mortality country profiles. WHO. 2020. Available from: Available from: http://www.who.int/gho/maternal_health/countries/en/. [cited cited 2020 Jul 2].

[28] UNICEF (2020). Key demographic indicators.

[29] DHS Program. Avaialble at https://dhsprogram.com/What-We-Do/Survey-Types/DHSMethodology.cfm#CP_JUMP_16156 Accessed on 27 Apr 2020.

[30] (ISTEEBU) IdSedÉÉdB, (MSPLS) MdlSPedlLclSB, International I (2012). Enquête Démographique et de Santé Burundi 2010.

[31] (MPBGP) MàlPcdlBGedPB, (MSPLS) MdlSPedlLclSB, (ISTEEBU) IdSedÉÉdB, ICF (2017). Troisième Enquête Démographique et de Santé.

[32] von Elm E, Altman DG, Egger M, Pocock SJ, Gøtzsche PC, Vandenbroucke JP (2014). The strengthening the reporting of observational studies in epidemiology (STROBE) statement: guidelines for reporting observational studies. Int J Surg.

[33] The World Bank. Overview. Updated on Nov 20, 2019. Retrieved from: https://www.worldbank.org/en/country/burundi/overview, http://www.worldbank.org/en/country/burundi/overview%3e. Accessed on 29 Apr 2020.

[34] The world Fact book. Central Intelligence Agency. Field listing: Literacy. Available from: https://www.cia.gov/library/publications/the-world-factbook/rankorder/2091rank.html, http://www.cia.gov/library/publications/the-world-factbook/rankorder/2091rank.html%3e. Accessed on 29 Apr 2020].

[35] Betrán AP, Temmerman M, Kingdon C, Mohiddin A, Opiyo N, Torloni MR (2018). Interventions to reduce unnecessary caesarean sections in healthy women and babies. Lancet.

